# Iron Consumption and Colorectal Cancer in Korean Adults: A Prospective Cohort Study

**DOI:** 10.3390/nu17081309

**Published:** 2025-04-09

**Authors:** Sukhong Min, Katherine De la Torre, Hyobin Lee, Woo-Kyoung Shin, Daehee Kang

**Affiliations:** 1 Department of Preventive Medicine, Seoul National University College of Medicine, Seoul 03080, Republic of Korea; sukhongmin@snu.ac.kr (S.M.); ktdelatorre@snu.ac.kr (K.D.l.T.); globalbin@snu.ac.kr (H.L.); 2 Department of Biomedical Sciences, Seoul National University Graduate School, Seoul 03080, Republic of Korea; 3 Integrated Major in Innovative Medical Science, Seoul National University Graduate School, Seoul 03080, Republic of Korea; 4 Division of Food and Pharmaceutical Technology, College of Health and Safety Science, Mokwon University, Daejeon 35349, Republic of Korea; shinwk@mokwon.ac.kr

**Keywords:** colorectal cancer, colon cancer, rectal cancer, iron, heme, diet, prospective studies

## Abstract

**Background/Objectives**: Colorectal cancer (CRC) is a major health concern in Korea, with its increasing incidence emphasizing the urgent need to identify risk factors. Recent studies suggest that heme iron elevates CRC risk, but evidence remains conflicting. This study examined the associations between total, heme, and non-heme iron intake and the incidence of colorectal, colon, and rectal cancer in Koreans. **Methods**: Using the Korean Genome and Epidemiology Study Health Examinee (KoGES HEXA) cohort, a large community-based cohort of healthy Koreans, we constructed a database of iron content for foods listed in a validated food frequency questionnaire (FFQ) and assessed dietary iron intake for each participant. Colorectal, colon, and rectal cancer cases were identified via the national cancer registry up to 2018. The association between iron consumption and cancers was evaluated with hazard ratios (HRs) and 95% confidence intervals (95%CIs) using multivariable-adjusted Cox regression. **Results**: During the 9.1-year median follow-up of 109,908 participants (37,697 men and 71,401 women, median age: 53.8 years), 608 new CRC cases were identified. Moderate total iron consumption in the second quintile (5.00–6.27 mg/day) decreased CRC (HR: 0.75; 95%CI: 0.58–0.97) and colon cancer (HR: 0.71; 95%CI: 0.51–1.00) risk compared to the lowest consumption quintile (1.09–4.99 mg/day), as did non-heme iron intake in the second quintile (4.98–6.24 mg/day) compared to its lowest quintile (1.09–4.97 mg/day) (CRC HR: 0.75; 95%CI: 0.58–0.98; colon cancer HR: 0.70; 95%CI: 0.49–0.98). **Conclusions**: Moderate total and non-heme iron intake reduced colorectal and colon cancer risk in Koreans, possibly via the displacement of carcinogens and the increased intake of protective micronutrients from plant-based foods. Larger-scale studies may be instrumental in substantiating these results.

## 1. Introduction

Colorectal cancer (CRC) remains a significant global health challenge, ranking as the third most common cancer among men and second among women, with over 1.9 million new cases diagnosed in 2020 [1]. In Korea, CRC has the second highest incidence and has been on the rise at an alarming rate of 2.6% per year since 2019 [2]. As such, the precise identification of risk factors for CRC is critical and urgently needed to form effective prevention strategies.

The multifactorial etiology of CRC includes modifiable risk factors such as smoking, excessive alcohol intake, physical inactivity, obesity, and dietary habits [3,4,5]. Two relatively recent additions are red and processed meat, classified by the World Cancer Research Fund (WCRF) and the International Agency for Research on Cancer (IARC) as risk factors for CRC in 2018 [6,7]. A proposed pathway from red and processed meat consumption to CRC incidence implicates heme iron. Studies have suggested that heme iron catalyzes the production of hydroxyl free radicals, apparent total *N*-nitroso compounds, and lipid peroxide radicals, all of which increase CRC risk [8,9].

Population studies, however, have shown inconsistent evidence linking heme iron intake to CRC risk with variations often observed across populations. Several studies from Western countries, including the USA and the Netherlands, have reported increased risks of colorectal, colon, and rectal cancer associated with heme iron [10,11,12]. However, Kabat et al. found its effect to be limited to the rectum [13]. Some studies have found that these associations are gender-specific; Aglago et al. found an increased risk of cancer only in men based on data from multiple European countries [14], while Zhang et al. found risks were increased only for women based on data from the USA [15]. In contrast to these Western findings, Hara et al. reported a decrease in the risk of these cancers associated with heme iron consumption among the Japanese population, though the results were not statistically significant [16].

Given the variability of these findings, it is also critical to consider the ethnic dietary contexts in which they occur. Notably, previous research has predominantly focused on Western populations, whose dietary patterns and genetic predispositions may differ significantly from those of Asian populations. Given that Asian diets are characterized by a higher consumption of vegetables, fish, and poultry and a lower intake of red meat and processed meat [17] and that epidemiological associations may vary by racial and ethnic groups [18,19], it remains to be researched whether epidemiological results from Western populations are generalizable to Asian contexts.

To address this gap, we explored the relationship between total iron, heme iron, and non-heme iron consumption and the incidence of CRC in a large cohort of healthy Koreans, hypothesizing that the associations may resemble findings from Asian populations more closely than those reported in Western populations, given similarities in dietary patterns and lifestyle factors.

## 2. Materials and Methods

### 2.1. Study Design and Population

The Korean Genome and Epidemiology Study’s Health Examinees cohort (KoGES HEXA) is a large-scale, community-based, prospective cohort study conducted in health examination centers and general hospitals throughout Korea. The rationale, design, and baseline characteristics of this study have been detailed previously [20]. Briefly, a baseline survey was conducted from 2004 to 2013 in which staff trained in computer-assisted personal interviewing administered structured questionnaires to interview HEXA participants on sociodemographic status, lifestyle factors, disease history and dietary intake and conducted physical examinations.

The present study used the Health Examinees-Gem (HEXA-G) participant eligibility criteria, where participants under 40 years or over 69 years of age, as well as those recruited from sites that only participated in the pilot phase, participated for less than 2 years, or did not meet the biospecimen quality control criteria, were excluded (*n* = 139,267). Further details were described in a previous study [21].

**Figure 1:**
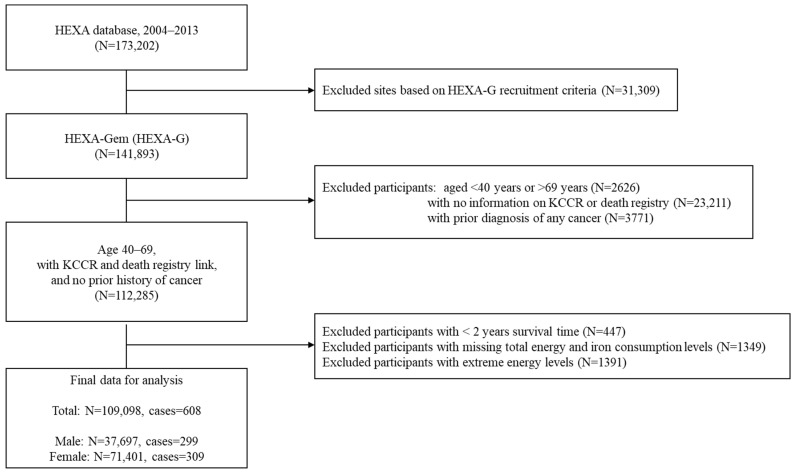
Flowchart for the overall selection process. In accordance with the Health Examinees-Gem (HEXA-G) participant eligibility criteria, participants under 40 years or over 69 years of age, as well as those recruited from sites that only participated in the pilot phase, participated for less than 2 years, or did not meet the biospecimen quality control criteria, were excluded (*n* = 139,267). Individuals missing mortality data or cancer registry data (*n* = 23,211), individuals with any prior history of cancer (*n* = 3771), those with follow-up periods of less than 2 years (*n* = 447), and those with missing dietary data (*n* = 1349) or reporting unreasonable total energy consumption values, defined as <800 or >4200 kcal/day for men and <500 or >3500 kcal/day for women, were also excluded (*n* = 1391).

For this study, we also excluded individuals missing mortality data or cancer registry data (*n* = 23,211), or those with any prior history of cancer (*n* = 3771). Participants with follow-up periods of less than 2 years (*n* = 447) were also excluded to avoid possible reverse causality. Participants missing dietary data (*n* = 1349) or reporting unreasonable total energy consumption values, defined as <800 or >4200 kcal/day for men and <500 or >3500 kcal/day for women, were also excluded (*n* = 1391) (Figure 1).

### 2.2. Exposure Assessment

At baseline, the participants’ usual dietary intake during the prior year was assessed via interviews using a semi-quantitative food frequency questionnaire (FFQ), which collected information on consumption frequency split into nine categories (almost never, once a month, two or three times a month, once or twice a week, three or four times a week, five or six times a week, once every day, twice every day, and three times a day) and consumption quantities divided into three categories (one-half standard serving, one standard serving, and one and a half servings) for 106 food items selected based on previous surveys and studies on the Korean diet. The development, reliability and validity of the KoGES HEXA FFQ are detailed elsewhere [22].

Based on the KoGES HEXA FFQ, a database of dietary iron content for each food item was constructed using a food composition table developed by the Korean Rural Development Administration (RDA) [23] and, where relevant data were missing, from the U.S. Department of Agriculture FoodData Central search results [24].

For each participant, the daily intake quantity of each food item was calculated using the reported frequency and quantity, then translated to iron consumption quantity, referred to as “total iron” hereafter. Heme iron intake was quantified based on previous research on the proportion of heme iron out of total iron in a given food source: 0.65 for beef, 0.39 for pork, 0.54 for processed meats, 0.26 for iron from poultry, fish, and shellfish, and 0.43 for all other meat sources of iron [11,12,14]. Other food sources were considered to lack heme iron. Non-heme iron intake was determined by subtracting the heme iron quantity from the total iron consumption.

Total iron and non-heme iron intake was categorized by quintiles to maximize exposure resolution while ensuring sufficient statistical power in each group. Heme iron, however, was categorized into quartiles as the absolute level of heme iron intake was too low for quintile analysis.

### 2.3. Outcome Ascertainment

The ascertainment of colorectal cancer cases was conducted through linkage to the Korea Central Cancer Registry, provided by the National Cancer Center of Korea, using the unique national identification number of each participant. The registry provided the type of cancer in International Classification of Diseases, 10th revision (ICD-10) codes and the date of diagnosis, until 31 December 2018. In this study, colon cancer was defined as C18, rectal cancer was defined as C20, and colorectal cancer was defined as C18, C19, and C20. Death certificate data collected up to 31 December 2019, provided by the Korean National Statistical Office, were similarly linked to KoGES HEXA data. Overall, person-years of follow-up were determined from the time of enrolment until the date of colorectal, colon, or rectal cancer diagnosis, the date of death, or the end of follow-up (31 December 2018), whichever was the earliest.

### 2.4. Covariates

Data on each participant’s age, sex, smoking status (never, former, current), alcohol consumption status (never, former, current), educational level (middle school, high school, college degree), family history of colorectal cancer among one’s parents, siblings, or offspring (yes, no), past history of hypertension (yes, no), past history of diabetes (yes, no), past history of hyperlipidemia (yes, no), and physical activity (exercise regularly, do not exercise regularly) were obtained by questionnaires at baseline.

Weight and height were obtained during physical examinations, from which the body mass index (BMI) was calculated as weight in kilograms divided by height in meters squared (kg/m^2^). It was then categorized into underweight (<18.5 kg/m^2^), normal weight (≥18.5, <23.0 kg/m^2^), overweight (≥23.0, <25.0 kg/m^2^), obese class I (≥25.0, <30.0 kg/m^2^), and obese class II (≥30.0 kg/m^2^), according to the Asian–Pacific classifications [25].

Similarly to how iron consumption was determined, total energy intake was estimated by multiplying the frequency and quantity of each food consumed listed in the KoGES HEXA FFQ and further multiplying that by the nutrient content stated in the aforementioned food composition table [23].

For covariates with missing values, constituting less than 5% for each variable, imputation was carried out using the median for continuous variables and the mode for categorical variables.

### 2.5. Statistical Analysis

The baseline characteristics of the study population were summarized using percentages for categorical variables and the mean, median, and standard deviation (SD) for continuous variables. The chi-square test was employed to compare baseline characteristics across total iron consumption categories for categorical variables. Analysis of variance was used for continuous variables.

Hazard ratios (HRs) and 95% confidence intervals (95%CIs) were calculated using Cox proportional hazards regression. For time scale, age at follow-up was used to account for left truncation of age. Entry time was the age at baseline recruitment, and exit time was the age at first colorectal cancer diagnosis, death, or the end of the follow-up period (31 December 2018), whichever occurred first.

Two models were constructed. For each model, we carried out the analysis with total iron, heme iron, and non-heme iron as exposure variables and colorectal cancer, colon cancer, and rectal cancer as outcome variables. Model 1 was adjusted for age and sex, and Model 2 was additionally adjusted for smoking, drinking, educational status, family history of colorectal cancer, past history of hypertension, past history of diabetes, past history of hyperlipidemia, physical activity, BMI, and total energy consumption. Additionally, for each model and for each type of iron, a linear trend between iron consumption and cancer risk was investigated. After analyses of the study population, subgroup analyses were performed with subgroups of men and women.

For all analyses, *p*-values were two-sided and considered statistically significant if less than 0.05. Analyses were performed using SAS 9.4 (SAS Institute, Cary, NC, USA; RRID: SCR_008567).

## 3. Results

In total, 109,908 participants (37,697 men; 71,401 women) were followed up for a median duration of 9.1 years for analysis, during which 608 new cases of CRC (299 men, 309 women) were newly diagnosed. Table 1 presents a summary of the baseline characteristics of the participants, categorized by total iron consumption quintiles. The mean age of the study participants was 53.8 years. Participants consuming the most iron, in Q5, tended to be men and younger, have a higher BMI and a higher level of education, and have experience smoking or drinking compared to those who consumed the least in Q1. Individuals in the Q1 group were more likely than those in Q5 to not exercise and have prior history of hypertension, diabetes, or dyslipidemia or a family history of colorectal cancer. On the other hand, the Q5 group consumed more total energy.

### 3.1. Association Between Total Iron Consumption and Cancer Risk

We found that total iron consumption per day in Q2 confers a statistically significant lower risk of CRC compared to the lowest quintile, Q1, in the fully adjusted Model 2. (HR: 0.75; 95%CI: 0.58–0.97) (Table 2). For colon cancer, Q2 showed a statistically significant lower risk (HR: 0.71; 95%CI: 0.51–1.00) as well as a significant trend (*p*-trend: 0.03). No significant associations were observed between rectal cancer and total iron consumption. Similar results, where Q2 showed a statistically significant lower risk of CRC (HR: 0.68; 95%CI: 0.47–0.99) and colon cancer (HR: 0.56; 95%CI: 0.34–0.93) compared to the Q1 group, were observed among men (Supplementary Table S1). While the risk for these cancers were also the lowest in Q2 for women (CRC HR: 0.80; 95%CI: 0.55–1.14; colon cancer HR: 0.85; 95%CI: 0.53–1.34), they were not statistically significant (Supplementary Table S2).

### 3.2. Association Between Heme Iron Consumption and Cancer Risk

Heme iron intake did not significantly affect CRC and colon cancer risk and did not show a linear trend (Table 3). While the highest heme iron intake group, Q4, showed decreased risk for rectal cancer compared to Q1, the difference was not statistically significant (HR: 0.81; 95%CI: 0.50–1.31). Subgroup analyses by gender also did not show any statistically significant results (Supplementary Tables S3 and S4).

### 3.3. Association Between Non-Heme Iron Consumption and Cancer Risk

We observed that the Q2 group for non-heme iron consumption showed a statistically significantly lower CRC risk compared to Q1 (HR: 0.75; 95%CI: 0.58–0.98) (Table 4), as well as a lower risk for colon cancer (HR: 0.70; 95%CI: 0.49–0.98). A significant trend was observed for colon cancer with increasing non-heme iron intake (*p*-trend: 0.04). No significant associations were observed between rectal cancer and non-heme iron consumption. For men, CRC and colon cancer risk was significantly reduced in the Q2 group compared to the Q1 group (CRC HR: 0.68; 95%CI: 0.47–0.99; colon cancer HR: 0.56; 95%CI: 0.34–0.92) but not for rectal cancer (Supplementary Table S5). For women, while the Q2 group showed the lowest risks for CRC (HR: 0.80; 95%CI: 0.56–1.16) and colon cancer (HR: 0.82; 95%CI: 0.51–1.30), the results were not significant (Supplementary Table S6).

## 4. Discussion

In this prospective cohort study, approximately 110,000 healthy Korean men and women were followed for a median of 9.1 years, during which 608 new CRC cases were diagnosed. The observed incidence was 60.5 cases per 100,000 person-years, which is approximately in line with national cancer statistics of Korea, where the crude incidence rate is 63.8 cases per 100,000 persons per year [26], supporting the representativeness of our cohort.

In this context, complex relationships between iron intake and colorectal, colon, and rectal cancer were observed. For total iron and non-heme iron, a statistically significant lower risk of CRC and colon cancer, but not rectal cancer, was observed in Q2 compared to Q1. CRC and colon cancer risks were also lower in higher total iron consumption quintiles and non-heme iron consumption quintiles than in Q1, although statistical significance was lost.

Previous studies that have explored the effects of total and non-heme iron consumption on colorectal cancer have reported mixed findings. Aglago et al. found that while total iron and non-heme iron reduced colorectal, colon, and rectal cancer risk among men, they increased the risk of these cancers for women [14]. Cross et al., who did not explore the effects of non-heme iron, similarly found total iron to decrease colorectal, colon, and rectal cancer risk [12]. On the other hand, Balder et al., Kabat et al., and Zhang et al. found that total iron increased this risk [11,13,15]. Meng et al. conducted a meta-analysis study of nine studies and found that while total iron did increase the risk of colorectal (OR: 1.01; 95%CI: 0.87–1.16), colon (OR: 1.42; 95%CI: 0.87–1.97), and rectal cancer (OR: 1.04; 95%CI: 0.67–1.42), the number of studies considered and the calculated pooled effect sizes were relatively small [27].

The previous literature does not offer definite biochemical pathways for the protective effect conferred by total iron or non-heme iron, as observed in this study. However, one possible explanation is that non-heme iron, primarily derived from plant-based foods such as legumes, vegetables, fruits, and whole grains, may exert protective effects indirectly. Previous studies have also suggested that micronutrients like fiber and chlorophylls reduce the risk of colorectal cancer by improving gut microbiota composition, reducing oxidative stress, and binding potential carcinogens [11,13]. In contrast, heme iron, mainly found in red meats, may promote colorectal carcinogenesis by facilitating the formation of carcinogenic *N*-nitroso compounds and increasing lipid peroxidation [8,9,14]. The observed protective effect of non-heme iron may reflect both the lower intake of harmful heme iron and the protective effects of plant-based foods rich in non-heme iron. Residual confounding effects, such that those who consume more non-heme iron, abundant in vegetable, fruits, beans, and leafy greens, tend to take more health-conscious actions not captured in our covariates, may also be responsible for its protective effects. Further research is required to provide evidence and elucidate the mechanism between total iron, non-heme iron, and colorectal cancer.

It is also worth noting that the protective effects of total iron or non-heme iron consumption, where observed, did not have a linear relationship and instead showed the greatest effect in Q2. This may be due to the generally low iron consumption levels among Koreans. While the recommended dietary allowance for iron is 8 mg for men and 18 mg and 8 mgs for women under 50 years and 51 years or older, respectively [28,29], and previous papers have reported the lowest iron consumption quintiles to have median intakes of 9.3 mg for men and 7.8 mgs for women [14], our lowest iron consumption quintile, Q1, ranged from 1.09 to 5.00 mgs, and our study did not show comparable levels of iron consumption until Q4. Low iron consumption has also been connected to increased cancer risk. Iron deficiency is connected to DNA replication stress and genome instability, defective heme biosynthesis and hypoxia, and suppression of tumor suppressor genes, as well as impaired immune response to tumors [30,31]. The reduced CRC and colon cancer risk observed in Q2, therefore, may reflect the increased risk of consuming inadequate levels of iron in Q1.

Heme iron appeared to have little influence over the three types of cancers. Despite the IARC’s decision [6], a number of studies also found no significant association between heme iron and CRC [13,14,15,27]. Hara et al., for one, reported from the Japan Public Health Center study that no significant associations were found between heme iron intake and CRC, and they attributed their observations to variations in dietary sources of heme iron and the overall Japanese diet’s composition [16]. Considering the dietary composition observed in this study, it is also possible that the difference in dietary composition produced the null association between heme iron and CRC. For Koreans, the consumption of iron and the more bioavailable heme iron may be too low overall, and as such, there may be a lack of biological difference between the quintiles used in this analysis. For example, while the recommended dietary allowance of iron is 10 mg/day for men and 18 mg/day for women [26], in this study, all quintiles except Q5 were far below this level.

Differences in iron consumption in the context of dietary consumption and composition may explain the differences in associations between Western populations and Asian populations. Dietary surveys suggest the average daily total iron intake among people in Japan and Korea to be 6.5 mg/day and 9.7 mg/day, respectively [32,33]. The total iron consumption level in China has been found to be much greater at 17.7 mg/day, but this is suggested to be mainly non-heme iron (16.2 mg/day) [34]. In contrast, the total iron intake in Western populations is typically higher—ranging from 10 to 22 mg/day—with approximately 11% consumed in the form of heme iron [35]. These differences in both iron quantity and source may influence CRC risk.

Subgroup analysis results by gender generally agreed with those of general population analyses, where the lowest risks for CRC and colon cancer were observed in Q2. For women, however, the results were not significant, which may be explained by the influence of sex hormones, particularly estrogen. Estrogen is thought to protect the colonic epithelium by restraining cell proliferation, enhancing DNA repair, reducing oxidative stress through the downregulation of NADPH oxidase activity, and limiting pro-carcinogenic inflammation [36,37]. It also promotes the proliferation of beneficial gut microbiota, further reducing colorectal carcinogenesis [36,38]. Collectively, these protective effects may reduce CRC incidence in women, resulting in lower statistical power to detect modest dietary effects. Nonetheless, our findings suggest that moderate total and non-heme iron intake may still be protective in women, consistent with trends observed in men, although this could not be confirmed statistically in our analysis.

Some potential limitations of this study must be considered. First, our dietary assessment relies on self-reported FFQs, which, despite being validated for nutrient intake [22], may introduce measurement errors due to the inherent limitations of self-reporting and the lack of information on food types not included in the survey. However, the KoGES HEXA FFQ had the important advantage of reflecting the Korean diet, thus providing a more accurate assessment of iron intake for Koreans. Dietary intake was also only assessed at baseline, and potential changes in dietary habits over the follow-up period were not accounted for. Thus, the exposure assessment may not fully reflect actual long-term dietary patterns, potentially influencing the observed associations. In addition, we did not include medications, iron supplements, or other agents affecting iron metabolism due to incomplete or unavailable data. While some of these, notably calcium, may influence CRC risk, we expect their impact on our findings to be minimal, as their use is unlikely to differ systematically across iron intake quintiles. Furthermore, several of our hazard ratios presented relatively wide confidence intervals or marginally significant *p*-values, which may reflect variability due to sample size limitations. These limitations highlight the need for larger-scale studies with repeated dietary assessments to more accurately evaluate the relationship between dietary factors and colorectal cancer risk.

Nevertheless, the strengths of our study include its prospective cohort design, which minimizes the potential for recall bias often encountered in case–control studies. This design is particularly advantageous in our examination of the association between iron consumption and colorectal cancer incidence. This study also includes evidence of the relationship between iron consumption, red meat, and colorectal cancer incidence in an Asian setting, a population with distinctively different dietary patterns. Utilizing a large population-based cohort enhances the generalizability of our findings, while the high accuracy and completeness of cancer diagnoses, achieved through retrieval from the Korea Central Cancer Registry, ensure the reliability of our outcome measures. Moreover, our study’s ability to adjust for a variety of confounding factors, including the participants’ past medical history, colorectal cancer family history, and other dietary habits, strengthens the validity of our conclusions regarding iron consumption’s impact on colorectal cancer risk.

## 5. Conclusions

In conclusion, our study demonstrates that high total and non-heme iron intake reduces colorectal and colon cancer risk among Koreans. These findings suggest the need for further research into the mechanisms underlying these associations and the role of dietary patterns in modulating cancer risk.

## Figures and Tables

**Table 1:** Characteristics of the study population.

Characteristic	All Sample	Total Iron Consumption (mg/Day)										*p*-Value ^1^
		Q1 (1.09–4.99)		Q2 (5.00–6.27)		Q3 (6.28–7.60)		Q4 (7.61–9.54)		Q5 (9.55–47.6)		
		N	%	N	%	N	%	N	%	N	%	
Number of individuals	109,098	21,820	20.0	21,820	20.0	21,820	20.0	21,819	20.0	21,819	20.0	<0.001
Person-years	367,079,182	201,327		199,804		199,848		201,291		203,427		
Follow-up year, y (median)	9.1	9.2		9.1		9.1		9.1		9.2		
Sex												
Men	37,697	5823	15.4	7132	18.9	7772	20.6	8317	22.1	8653	23.0	<0.001
Women	71,401	15,997	22.4	14,688	20.6	14,048	19.7	13,502	18.9	13,166	18.4	
Age, y (mean (SD))	52.8 (8.0)	53.7 (7.9)		53.0 (8.0)		52.0 (8.0)		52.0 (8.0)		51.0 (7.9)		<0.001
BMI ^2^, kg/m^2^ (mean(SD))	23.9 (2.9)	23.9 (2.9)		23.9 (2.9)		23.9 (2.9)		24.0 (2.9)		24.1 (2.9)		<0.001
Underweight (<18.5)	1922	453	23.6	425	22.1	358	18.6	359	18.7	327	17.0	<0.001
Normal (≥18.5 and <23.0)	41,613	8694	20.9	8580	20.6	8260	19.8	8253	19.8	7826	18.8	
Overweight (≥23.0 and <25.0)	30,318	6018	19.8	5976	19.7	6112	20.2	6057	20.0	6155	20.3	
Obese I (≥25.0 and <30.0)	32,208	6113	19.0	6277	19.5	6493	20.2	6505	20.2	6820	21.2	
Obese II (≥30.0 kg/m^2^)	3037	542	17.8	562	18.5	597	19.7	645	21.2	691	22.8	
Education												
Middle school	32,963	8652	26.2	7248	22.0	6492	19.7	5659	17.2	4912	14.9	<0.001
High school	47,332	8720	18.4	9285	19.6	9439	19.9	9788	20.7	10,100	21.3	
College degree	28,803	4448	15.4	5287	18.4	5889	20.4	6372	22.1	6807	23.6	
Smoking												
Never	79,217	17,179	21.7	16,242	20.5	15,658	19.8	15,169	19.1	14,969	18.9	<0.001
Former	16,301	2533	15.5	3104	19.0	3489	21.4	3622	22.2	3553	21.8	
Current	13,580	2108	15.5	2474	18.2	2673	19.7	3028	22.3	3297	24.3	
Alcohol												
Never	55,383	12,453	22.5	11,456	20.7	10,860	19.6	10,518	19.0	10,096	18.2	<0.001
Former	3744	763	20.4	734	19.6	708	18.9	712	19.0	827	22.1	
Current	49,971	8604	17.2	9630	19.3	10,252	20.5	10,589	21.2	10,896	21.8	
Physical activity												
Do not regularly exercise	54,818	11,943	21.8	11,260	20.5	10,956	20.0	10,668	19.5	9991	18.2	<0.001
Regularly exercise, MIPA ^3^ < 150	12,956	2592	20.0	2634	20.3	2552	19.7	2468	19.0	2710	20.9	
Regularly exercise, MIPA ^3^ ≥ 150	41,324	7285	17.6	7926	19.2	8312	20.1	8683	21.0	9118	22.1	
History of hypertension												
Yes	20,405	4442	21.8	4221	20.7	4086	20.0	3976	19.5	3680	18.0	<0.001
No	88,693	17,378	19.6	17,599	19.8	17,734	20.0	17,843	20.1	18,139	20.5	
History of diabetes												
Yes	6965	1551	22.3	1452	20.8	1365	19.6	1258	18.1	1339	19.2	<0.001
No	102,133	20,269	19.8	20,368	19.9	20,455	20.0	20,561	20.1	20,480	20.1	
History of hyperlipidemia												
Yes	10,195	2219	21.8	2108	20.7	2017	19.8	1937	19.0	1914	18.8	<0.001
No	98,903	19,601	19.8	19,712	19.9	19,803	20.0	19,882	20.1	19,905	20.1	
Family history of colorectal cancer												
Yes	752	166	22.1	171	22.7	139	18.5	152	20.2	124	16.5	0.03
No	108,346	21,654	20.0	21,649	20.0	21,681	20.0	21,667	20.0	21,695	20.0	
Total energy consumption, kcal/day (mean, SD)	1694.7 (496.7)	1261.6 (276.5)		1536.3 (262.3)		1712.3 (283.2)		1913.0 (322.2)		2280.3 (475.3)		<0.001

^1^ Significance tests reflect differences across quintiles of total iron consumption based on analysis of variance (continuous variables) and the chi-square test (categorical variables). ^2^ BMI: body mass index. ^3^ MIPA: medium-intensity physical activity.

**Table 2:** Association between total iron consumption and colorectal, colon, and rectal cancer risk.

	Total Iron Consumption (mg/Day)					*p-*Trend
	Q1 (1.09–4.99)	Q2 (5.00–6.27)	Q3 (6.28–7.60)	Q4 (7.61–9.54)	Q5 (9.55–47.6)	
	HR (95%CI)	HR (95%CI)	HR (95%CI)	HR (95%CI)	HR (95%CI)	
Person-years	201,327	199,804	199,848	201,291	203,427	
N	21,820	21,820	21,820	21,819	21,819	
Colorectal cancer						
Cases	143	107	110	136	112	
Model 1 ^1^	1.00 (ref)	0.75 (0.59–0.97)	0.78 (0.61–1.00)	0.97 (0.76–1.23)	0.80 (0.62–1.02)	0.07
Model 2 ^2^	1.00 (ref)	0.75 (0.58–0.97)	0.77 (0.59–1.01)	0.96 (0.72–1.28)	0.79 (0.55–1.13)	0.08
Colon cancer						
Cases	40	26	32	43	33	
Model 1 ^1^	1.00 (ref)	0.71 (0.51–0.99)	0.75 (0.54–1.03)	1.11 (0.83–1.50)	0.91 (0.67–1.25)	0.03
Model 2 ^2^	1.00 (ref)	0.71 (0.51–1.00)	0.75 (0.53–1.07)	1.12 (0.79–1.61)	0.93 (0.59–1.46)	0.03
Rectal cancer						
Cases	47	34	30	48	41	
Model 1 ^1^	1.00 (ref)	0.84 (0.55–1.29)	0.89 (0.58–1.36)	0.78 (0.50–1.20)	0.66 (0.42–1.05)	0.48
Model 2 ^2^	1.00 (ref)	0.84 (0.54–1.32)	0.90 (0.56–1.43)	0.79 (0.46–1.33)	0.68 (0.35–1.31)	0.82

^1^ Model 1: adjusted for age and sex. ^2^ Model 2: additionally adjusted for smoking (never/former/current), drinking (never/former/current), educational status (middle school, high school, college degree), family history of colorectal cancer (yes/no), past history of hypertension (yes/no), past history of diabetes (yes/no), past history of hyperlipidemia (yes/no), physical activity level (do not regularly exercise, regularly exercise with MIPA < 150, regularly exercise with MIPA ≥ 150), BMI (<18.5 kg/m^2^, ≥18.5 and <23.0 kg/m^2^, ≥23.0 and <25.0 kg/m^2^, ≥25.0 and <30.0 kg/m^2^, ≥30.0 kg/m^2^), and total energy consumption.

**Table 3:** Association between heme iron consumption and colorectal, colon, and rectal cancer risk.

	Heme Iron Consumption (mg/Day)				*p-*Trend
	Q1 (0.00–0.02)	Q2 (0.03–0.04)	Q3 (0.05–0.06)	Q4 (0.07–0.68)	
	HR (95%CI)	HR (95%CI)	HR (95%CI)	HR (95%CI)	
Person-years	252,608	251,361	251,815	249,912	
N	27,274	27,277	27,273	27,274	
Colorectal cancer					
Cases	156	153	153	146	
Model 1 ^1^	1.00 (ref)	1.01 (0.81–1.26)	1.03 (0.82–1.29)	0.98 (0.78–1.23)	0.97
Model 2 ^2^	1.00 (ref)	1.01 (0.80–1.26)	1.04 (0.82–1.31)	0.99 (0.77–1.29)	0.98
Colon cancer					
Cases	98	93	85	98	
Model 1 ^1^	1.00 (ref)	1.00 (0.75–1.33)	0.95 (0.71–1.28)	1.11 (0.83–1.47)	0.78
Model 2 ^2^	1.00 (ref)	0.99 (0.74–1.32)	0.94 (0.69–1.28)	1.09 (0.79–1.50)	0.82
Rectal cancer					
Cases	48	53	56	38	
Model 1 ^1^	1.00 (ref)	1.08 (0.73–1.60)	1.12 (0.76–1.66)	0.74 (0.48–1.14)	0.21
Model 2 ^2^	1.00 (ref)	1.11 (0.75–1.66)	1.19 (0.79–1.79)	0.81 (0.50–1.31)	0.31

^1^ Model 1: adjusted for age and sex. ^2^ Model 2: additionally adjusted for smoking (never/former/current), drinking (never/former/current), educational status (middle school, high school, college degree), family history of colorectal cancer (yes/no), past history of hypertension (yes/no), past history of diabetes (yes/no), past history of hyperlipidemia (yes/no), physical activity level (do not regularly exercise, regularly exercise with MIPA < 150, regularly exercise with MIPA ≥ 150), BMI (<18.5 kg/m^2^, ≥18.5 and <23.0 kg/m^2^, ≥23.0 and <25.0 kg/m^2^, ≥25.0 and <30.0 kg/m^2^, ≥30.0 kg/m^2^), and total energy consumption.

**Table 4:** Association between non-heme iron consumption and colorectal, colon, and rectal cancer risk.

	Non-Heme Iron Consumption (mg/Day)					*p-*Trend
	Q1 (1.09–4.97)	Q2 (4.98–6.24)	Q3 (6.25–7.56)	Q4 (7.57–9.48)	Q5 (9.49–47.53)	
	HR (95%CI)	HR (95%CI)	HR (95%CI)	HR (95%CI)	HR (95%CI)	
Person-years	201,319	199,742	199,883	201,296	203,456	
N	21,819	21,821	21,819	21,821	21,818	
Colorectal cancer						
Cases	142	107	113	136	110	
Model 1 ^1^	1.00 (ref)	0.76 (0.59–0.98)	0.81 (0.63–1.03)	0.97 (0.77–1.23)	0.79 (0.61–1.01)	0.09
Model 2 ^2^	1.00 (ref)	0.75 (0.58–0.98)	0.80 (0.61–1.04)	0.96 (0.72–1.28)	0.77 (0.54–1.11)	0.10
Colon cancer						
Cases	87	59	65	91	72	
Model 1 ^1^	1.00 (ref)	0.70 (0.50–0.97)	0.78 (0.57–1.08)	1.11 (0.83–1.50)	0.89 (0.65–1.22)	0.04
Model 2 ^2^	1.00 (ref)	0.70 (0.49–0.98)	0.78 (0.55–1.10)	1.11 (0.77–1.58)	0.88 (0.56–1.39)	0.04
Rectal cancer						
Cases	44	40	42	37	32	
Model 1 ^1^	1.00 (ref)	0.88 (0.57–1.35)	0.91 (0.60–1.39)	0.79 (0.51–1.23)	0.68 (0.43–1.07)	0.54
Model 2 ^2^	1.00 (ref)	0.89 (0.57–1.39)	0.92 (0.58–1.47)	0.81 (0.48–1.37)	0.71 (0.37–1.37)	0.87

^1^ Model 1: adjusted for age and sex. ^2^ Model 2: additionally adjusted for smoking (never/former/current), drinking (never/former/current), educational status (middle school, high school, college degree), family history of colorectal cancer (yes/no), past history of hypertension (yes/no), past history of diabetes (yes/no), past history of hyperlipidemia (yes/no), physical activity level (do not regularly exercise, regularly exercise with MIPA < 150, regularly exercise with MIPA ≥ 150), BMI (<18.5 kg/m^2^, ≥18.5 and <23.0 kg/m^2^, ≥23.0 and <25.0 kg/m^2^, ≥25.0 and <30.0 kg/m^2^, ≥30.0 kg/m^2^), and total energy consumption.

## Data Availability

Data described in the manuscript, code book, and analytic code will not be made available due to the inclusion of personal data that may potentially be sensitive to the patients, even though researchers are provided with an anonymized dataset that excludes resident registration numbers.

## Supplementary Materials

The following supporting information can be downloaded at https://www.mdpi.com/article/10.3390/nu17081309/s1: Table S1: Association between total iron consumption and colorectal, colon, and rectal cancer risk among men; Table S2: Association between total iron consumption and colorectal, colon, and rectal cancer risk among women; Table S3: Association between heme iron consumption and colorectal, colon, and rectal cancer risk for men; Table S4: Association between heme iron consumption and colorectal, colon, and rectal cancer risk for women; Table S5: Association between non-heme iron consumption and colorectal, colon, and rectal cancer risk among men; Table S6: Association between non-heme iron consumption and colorectal, colon, and rectal cancer risk among women.

## Abbreviations

The following abbreviations are used in this manuscript:

BMI	Body mass index
CI	Confidence interval
CRC	Colorectal cancer
FFQ	Food frequency questionnaire
HR	Hazard ratio
IARC	International Agency for Research on Cancer
ICD	International classification of diseases
KoGES HEXA	Korean Genome and Epidemiology Study Health Examinees
RDA	Rural Development Administration
SD	Standard deviation
WCRF	World Cancer Research Fund
