# Supplementary material for: Iron Consumption and Colorectal Cancer in Korean Adults: A Prospective Cohort Study
Source: Nutrients. 2025 Apr 9;17(8):1309. doi: 10.3390/nu17081309 (PMC12029972; doi:10.3390/nu17081309)
Supplement: Supplementary file 1 [file nutrients-17-01309-s001.zip › nutrients-3558248-supplementary.pdf]

Iron Consumption and Colorectal Cancer in Korean Adults: A Prospective Cohort Study
Sukhong Min

SUPPLEMENTARY DATA

Supplementary Table S1. Association between total iron consumption and colorectal, colon, and rectal cancer risk among men

	Total iron consumption (mg/day)					*p*-trend
	Q1 (1.09-4.99)	Q2 (5.00-6.27)	Q3 (6.28-7.60)	Q4 (7.61-9.54)	Q5 (9.55-47.6)	
	HR (95% CI)	HR (95% CI)	HR (95% CI)	HR (95% CI)	HR (95% CI)	
Person-years	53,988	65,754	71,210	76,301	80,086	
N	5,823	7,132	7,772	8,317	8,653	
Colorectal cancer						
Cases	68	53	55	68	55	
Model 1[1]	1.00 (ref)	0.66(0.46-0.95)	0.65(0.46-0.93)	0.78(0.55-1.09)	0.61(0.43-0.87)	0.04
Model 2[2]	1.00 (ref)	0.68(0.47-0.99)	0.69(0.47-1.02)	0.84(0.56-1.27)	0.70(0.42-1.18)	0.21
Colon cancer						
Cases	17	11	11	13	18	
Model 1[1]	1.00 (ref)	0.53(0.33-0.87)	0.47(0.28-0.77)	0.77(0.50-1.19)	0.58(0.37-0.93)	0.01
Model 2[2]	1.00 (ref)	0.56(0.34-0.93)	0.51(0.30-0.88)	0.89(0.53-1.51)	0.75(0.38-1.47)	0.04
Rectal cancer						
Cases	25	15	13	28	14	
Model 1[1]	1.00 (ref)	0.92(0.51-1.67)	0.98(0.55-1.75)	0.87(0.48-1.56)	0.70(0.38-1.29)	0.78
Model 2[2]	1.00 (ref)	0.95(0.52-1.75)	1.04(0.56-1.95)	0.94(0.47-1.89)	0.81(0.34-1.92)	0.97

[1] Model 1: Adjusted for age

[2] Model 2: Additionally adjusted for smoking (never/former/current), drinking (never/former/current), educational status (middle school, high school, college degree), family history of colorectal cancer (yes/no), past history of hypertension (yes/no), past history of diabetes (yes/no), past history of hyperlipidemia (yes/no), physical activity level (do not regularly exercise, regularly exercise with MIPA <150, regularly exercise with MIPA≥150), BMI (<18.5 kg/m^2, ≥18.5 and <23.0 kg/m^2, ≥23.0 and <25.0 kg/m^2, ≥25.0 and <30.0 kg/m^2, ≥30.0 kg/m^2), and total energy consumption

Supplementary Table S2. Association between total iron consumption and colorectal, colon, and rectal cancer risk among women

	Total iron consumption (mg/day)					
	Q1 (1.09-4.99)	Q2 (5.00-6.27)	Q3 (6.28-7.60)	Q4 (7.61-9.54)	Q5 (9.55-47.6)	*p*-trend
	HR (95% CI)	HR (95% CI)	HR (95% CI)	HR (95% CI)	HR (95% CI)	
Person-years	147,339	134,051	128,637	124,990	123,341	
N	15,997	14,688	14,048	13,502	13,166	
Colorectal cancer						
Cases	75	54	55	68	57	
Model 1[1]	1.00 (ref)	0.83(0.58-1.18)	0.90(0.64-1.27)	1.17(0.84-1.63)	1.02(0.72-1.44)	0.38
Model 2[2]	1.00 (ref)	0.80(0.55-1.14)	0.84(0.57-1.24)	1.07(0.72-1.60)	0.89(0.54-1.47)	0.45
Colon cancer						
Cases	23	15	21	30	15	
Model 1[1]	1.00 (ref)	0.88(0.56-1.37)	1.05(0.68-1.62)	1.46(0.97-2.19)	1.28(0.84-1.95)	0.14
Model 2[2]	1.00 (ref)	0.85(0.53-1.34)	0.99(0.62-1.59)	1.34(0.83-2.19)	1.13(0.62-2.06)	0.38
Rectal cancer						
Cases	22	19	17	20	27	
Model 1[1]	1.00 (ref)	0.76(0.40-1.42)	0.80(0.42-1.50)	0.67(0.34-1.32)	0.64(0.32-1.29)	0.70
Model 2[2]	1.00 (ref)	0.73(0.38-1.42)	0.75(0.37-1.53)	0.63(0.28-1.43)	0.57(0.21-1.57)	0.81

[1] Model 1: Adjusted for age

[2] Model 2: Additionally adjusted for smoking (never/former/current), drinking (never/former/current), educational status (middle school, high school, college degree), family history of colorectal cancer (yes/no), past history of hypertension (yes/no), past history of diabetes (yes/no), past history of hyperlipidemia (yes/no), physical activity level (do not regularly exercise, regularly exercise with MIPA <150, regularly exercise with MIPA≥150), BMI (<18.5 kg/m^2, ≥18.5 and <23.0 kg/m^2, ≥23.0 and <25.0 kg/m^2, ≥25.0 and <30.0 kg/m^2, ≥30.0 kg/m^2), and total energy consumption

Supplementary Table S3. Association between heme iron consumption and colorectal, colon, and rectal cancer risk for men

	Heme iron consumption (mg/day)				
	Q1 (0.00-0.02)	Q2 (0.03-0.04)	Q3 (0.05-0.06)	Q4 (0.07-0.68)	*p*-trend
	HR (95% CI)	HR (95% CI)	HR (95% CI)	HR (95% CI)	
Person-years	65,086	81,320	94,796	106,136	
N	7,028	8,819	10,282	11,568	
Colorectal cancer					
Cases	63	81	77	78	
Model 1[1]	1.00 (ref)	1.10(0.79-1.53)	0.96(0.69-1.34)	0.90(0.64-1.25)	0.62
Model 2[2]	1.00 (ref)	1.12(0.81-1.57)	1.03(0.73-1.47)	1.02(0.70-1.49)	0.90
Colon cancer					
Cases	40	43	37	45	
Model 1[1]	1.00 (ref)	0.93(0.60-1.43)	0.74(0.47-1.17)	0.84(0.55-1.29)	0.59
Model 2[2]	1.00 (ref)	0.95(0.61-1.47)	0.80(0.50-1.27)	0.95(0.58-1.55)	0.77
Rectal cancer					
Cases	20	35	32	27	
Model 1[1]	1.00 (ref)	1.48(0.85-2.56)	1.22(0.70-2.14)	0.94(0.53-1.68)	0.29
Model 2[2]	1.00 (ref)	1.52(0.87-2.65)	1.33(0.74-2.38)	1.08(0.56-2.06)	0.39

[1] Model 1: Adjusted for age

[2] Model 2: Additionally adjusted for smoking (never/former/current), drinking (never/former/current), educational status (middle school, high school, college degree), family history of colorectal cancer (yes/no), past history of hypertension (yes/no), past history of diabetes (yes/no), past history of hyperlipidemia (yes/no), physical activity level (do not regularly exercise, regularly exercise with MIPA <150, regularly exercise with MIPA≥150), BMI (<18.5 kg/m^2, ≥18.5 and <23.0 kg/m^2, ≥23.0 and <25.0 kg/m^2, ≥25.0 and <30.0 kg/m^2, ≥30.0 kg/m^2), and total energy consumption

Supplementary Table S4. Association between heme iron consumption and colorectal, colon, and rectal cancer risk for women

	Heme iron consumption (mg/day)				p-trend
	Q1 (0.00-0.02)	Q2 (0.03-0.04)	Q3 (0.05-0.06)	Q4 (0.07-0.68)	
	HR (95% CI)	HR (95% CI)	HR (95% CI)	HR (95% CI)	
Person-years	187,522	170,041	157,019	143,776	
N	20,246	18,458	16,991	15,706	
Colorectal cancer					
Cases	93	72	76	68	
Model 1[1]	1.00 (ref)	0.92(0.67-1.25)	1.10(0.81-1.49)	1.07(0.78-1.47)	0.69
Model 2[2]	1.00 (ref)	0.89(0.65-1.22)	1.04(0.75-1.43)	0.98(0.69-1.40)	0.81
Colon cancer					
Cases	58	50	48	53	
Model 1[1]	1.00 (ref)	1.03(0.70-1.50)	1.14(0.77-1.67)	1.36(0.94-1.98)	0.38
Model 2[2]	1.00 (ref)	0.98(0.67-1.44)	1.04(0.70-1.56)	1.19(0.78-1.82)	0.81
Rectal cancer					
Cases	28	18	24	11	
Model 1[1]	1.00 (ref)	0.74(0.41-1.35)	1.11(0.64-1.92)	0.55(0.28-1.11)	0.20
Model 2[2]	1.00 (ref)	0.77(0.42-1.41)	1.15(0.64-2.06)	0.58(0.27-1.26)	0.25

[1] Model 1: Adjusted for age

[2] Model 2: Additionally adjusted for smoking (never/former/current), drinking (never/former/current), educational status (middle school, high school, college degree), family history of colorectal cancer (yes/no), past history of hypertension (yes/no), past history of diabetes (yes/no), past history of hyperlipidemia (yes/no), physical activity level (do not regularly exercise, regularly exercise with MIPA <150, regularly exercise with MIPA≥150), BMI (<18.5 kg/m^2, ≥18.5 and <23.0 kg/m^2, ≥23.0 and <25.0 kg/m^2, ≥25.0 and <30.0 kg/m^2, ≥30.0 kg/m^2), and total energy consumption

Supplementary Table S5. Association between non-heme iron consumption and colorectal, colon, and rectal cancer risk among men

	Non-heme iron consumption (mg/day)					
	Q1 (1.09-4.97)	Q2 (4.98-6.24)	Q3 (6.25-7.56)	Q4 (7.57-9.48)	Q5 (9.49-47.53)	*p*-trend
	HR (95% CI)	HR (95% CI)	HR (95% CI)	HR (95% CI)	HR (95% CI)	
Person-years	54,087	65,704	71,239	76,339	79,970	
N	5,835	7,127	7,775	8,320	8,640	
Colorectal cancer						
Cases	68	53	56	68	54	
Model 1[1]	1.00 (ref)	0.67(0.46-0.95)	0.66(0.47-0.95)	0.78(0.55-1.09)	0.60(0.42-0.86)	0.04
Model 2[2]	1.00 (ref)	0.68(0.47-0.99)	0.70(0.47-1.03)	0.83(0.56-1.26)	0.68(0.40-1.14)	0.21
Colon cancer						
Cases	42	26	25	41	31	
Model 1[1]	1.00 (ref)	0.53(0.33-0.87)	0.48(0.30-0.80)	0.77(0.50-1.19)	0.57(0.36-0.91)	0.02
Model 2[2]	1.00 (ref)	0.56(0.34-0.92)	0.53(0.31-0.90)	0.87(0.52-1.49)	0.70(0.36-1.39)	0.05
Rectal cancer						
Cases	21	23	26	24	20	
Model 1[1]	1.00 (ref)	0.93(0.51-1.67)	0.98(0.55-1.75)	0.87(0.48-1.56)	0.70(0.38-1.30)	0.79
Model 2[2]	1.00 (ref)	0.96(0.52-1.76)	1.04(0.56-1.96)	0.94(0.47-1.89)	0.81(0.34-1.94)	0.97

[1] Model 1: Adjusted for age

[2] Model 2: Additionally adjusted for smoking (never/former/current), drinking (never/former/current), educational status (middle school, high school, college degree), family history of colorectal cancer (yes/no), past history of hypertension (yes/no), past history of diabetes (yes/no), past history of hyperlipidemia (yes/no), physical activity level (do not regularly exercise, regularly exercise with MIPA <150, regularly exercise with MIPA≥150), BMI (<18.5 kg/m^2, ≥18.5 and <23.0 kg/m^2, ≥23.0 and <25.0 kg/m^2, ≥25.0 and <30.0 kg/m^2, ≥30.0 kg/m^2), and total energy consumption

Supplementary Table S6. Association between non-heme iron consumption and colorectal, colon, and rectal cancer risk among women

	Non-heme iron consumption (mg/day)					*p*-trend
	Q1 (1.09-4.97)	Q2 (4.98-6.24)	Q3 (6.25-7.56)	Q4 (7.57-9.48)	Q5 (9.49-47.53)	
	HR (95% CI)	HR (95% CI)	HR (95% CI)	HR (95% CI)	HR (95% CI)	
Person-years	147,232	134,038	128,644	124,957	123,486	
N	15,984	14,694	14,044	13,501	13,178	
Colorectal cancer						
Cases	74	54	57	68	56	
Model 1[1]	1.00 (ref)	0.84(0.59-1.19)	0.94(0.67-1.33)	1.19(0.85-1.65)	1.01(0.71-1.44)	0.43
Model 2[2]	1.00 (ref)	0.80(0.56-1.16)	0.88(0.60-1.29)	1.08(0.72-1.61)	0.88(0.53-1.45)	0.50
Colon cancer						
Cases	45	33	40	50	41	
Model 1[1]	1.00 (ref)	0.85(0.54-1.33)	1.10(0.72-1.69)	1.46(0.97-2.18)	1.25(0.81-1.91)	0.14
Model 2[2]	1.00 (ref)	0.82(0.51-1.30)	1.03(0.65-1.65)	1.33(0.81-2.16)	1.08(0.59-1.98)	0.36
Rectal cancer						
Cases	23	17	16	13	12	
Model 1[1]	1.00 (ref)	0.84(0.45-1.57)	0.83(0.44-1.57)	0.70(0.36-1.39)	0.67(0.33-1.35)	0.79
Model 2[2]	1.00 (ref)	0.82(0.42-1.58)	0.79(0.39-1.62)	0.67(0.30-1.53)	0.62(0.23-1.69)	0.89

[1] Model 1: Adjusted for age

[2] Model 2: Additionally adjusted for smoking (never/former/current), drinking (never/former/current), educational status (middle school, high school, college degree), family history of colorectal cancer (yes/no), past history of hypertension (yes/no), past history of diabetes (yes/no), past history of hyperlipidemia (yes/no), physical activity level (do not regularly exercise, regularly exercise with MIPA <150, regularly exercise with MIPA≥150), BMI (<18.5 kg/m^2, ≥18.5 and <23.0 kg/m^2, ≥23.0 and <25.0 kg/m^2, ≥25.0 and <30.0 kg/m^2, ≥30.0 kg/m^2), and total energy consumption
